# Care as Usual: An Acceptable Strategy to Apply During the COVID-19 Pandemic in a French Tertiary Gynecologic Oncology Department

**DOI:** 10.3389/fonc.2021.653009

**Published:** 2021-04-21

**Authors:** Guillaume Blache, Houssein El Hajj, Camille Jauffret, Gilles Houvenaeghel, Laura Sabiani, Julien Barrou, Isabelle Masquin, Jérémy Le Saout, Djamel Mokart, Marion Faucher, Eric Lambaudie

**Affiliations:** ^1^Department of Surgical Oncology, Paoli Calmettes Institute, Marseille, France; ^2^Department of surgical oncology, Institut Paoli-Calmettes and CRCM, CNRS, INSERM, Aix Marseille Université, Marseille, France; ^3^Department of Anesthesiology and Critical Care, Paoli Calmettes Institute, Marseille, France

**Keywords:** COVID 19 pandemic, patient centered clinical pathway, gynecologic oncology, surgical activity, surgical oncology

## Abstract

We describe and analyze a “care as usual” strategy of a French Comprehensive Cancer Center during the COVID-19 pandemic to manage surgical patients with gynecological cancer. We conducted a retrospective analysis evaluating the surgical activity in our gynecologic oncology department between January 21 and May 12, 2020. We compared the surgical activity and surgical and oncologic outcomes during the pre-lockdown period and the pandemic period. The main objective was to evaluate the impact of the COVID-19 pandemic on surgical activity. The secondary objectives were to analyze the surgical and the oncologic outcomes. We compared the surgical activity during the 8 weeks after the national lockdown (85 procedures) to the surgical activity in the 8 weeks preceding the lockdown (127 procedures). We observed a 33% decrease in activity between the two periods. The clinical and epidemiologic characteristics were similar between the two periods. There were no differences between the surgical approaches (*p* = 0.592), the surgical complexity (*p* = 0.323), the length of stay (*p* = 0.85), and even for the complex procedure (*p* = 0.96) and the perioperative (*p* = 0.791) and postoperative complication rates (*p* = 0.102). We observed a significant decrease in the time of return to intended oncological treatment (RIOT) during the lockdown period with an average of 31.9 days compared to 46.9 days in the pre-lockdown period (*p* = 0.003). During the COVID-19 pandemic, “care as usual” represents an acceptable strategy without impairing the oncologic outcome in a Comprehensive Cancer Center with a patient-centered clinical pathway for gynecologic oncologic surgical patients.

## Introduction

In 2019, a novel virus (SARS-CoV-2) inducing severe acute respiratory syndrome (COVID-19) has spread so fast around the world. This led the World Health Organization to declare it on January 31, 2020, as a public health emergency of international concern. France, just like the rest of the world, was impacted by this pandemic. The French authorities declared a nationwide lockdown between March 17 and May 11, 2020. This pandemic induced a serious negative impact on healthcare resources. Hospital beds, intensive care unit (ICU) beds, and mechanical ventilators were either occupied by or reserved for COVID-19 patients in the majority of the hospitals (1). This strategy resulted in reduced access to healthcare services for emergency department patients, patients with chronic diseases, and cancer patients.

Due to the immunosuppressive effect of the malignancy itself or secondary to oncologic treatments (surgery, chemotherapy, and radiotherapy), cancer patients are more susceptible and vulnerable to the COVID-19 infection than the rest of the population, resulting in a poorer prognosis (2). New recommendations have been issued by national and international scientific societies to adjust the clinical practice in gynecologic oncology to best adapt to the COVID-19 pandemic (3, 4). Surgical indications were reduced, and less aggressive therapies were recommended (3–6). The increased time interval between diagnosis and treatment imposed by the COVID-19 pandemic and the associated lockdown have negatively impacted the oncologic outcomes of the patients. Grass et al. (7) showed in their study that a shorter interval time between surgery and adjuvant treatment increased overall survival in patients.

However, Yang et al. (8) in their study at the Wuhan Cancer Center did not report any case of COVID-19 infections in gynecologic oncology patients who underwent surgery. These findings suggest that the strategy “care as usual” could be applied in specialized comprehensive cancer centers with a high volume of surgical patients. These centers developed patient-centered clinical pathways for each oncologic indication in association with Enhanced Recovery Surgery (ERAS) Protocols. The previously cited programs aim to improve postoperative surgical and oncological outcomes and particularly the return to intended oncological treatment (RIOT).

The primary objective of this study is to analyze the impact of the “care as usual” strategy on the surgical activity in the gynecologic oncology department of a comprehensive cancer center in France during the lockdown period. The secondary objectives are the analysis of surgical and oncologic outcomes.

## Methods

### Study Population

We conducted a retrospective analysis evaluating the surgical activity in the gynecologic oncology department at a comprehensive cancer center between January 21 and May 12, 2020. We compared the surgical activity and surgical and oncologic outcomes during two periods: The first period consisted of the last 8 weeks preceding the lockdown, and the second period consisted of the 8 weeks that followed the announcement of the lockdown.

All patients who underwent surgery for gynecologic malignancy were included in the study regardless of their time of diagnosis or follow-up. Patients who were not treated surgically were excluded from the study. This study was performed following the precepts established by the Helsinki declaration and validated by our cancer center ethical committee.

For every patient treated, the following data were collected: patient's age, body mass index (BMI), ASA score, the type of surgery (simple diagnostic procedure or complex therapeutic procedure), the length of hospital stay (LOS), the surgical approach (pelvic examination under anesthesia, hysteroscopy, minimally invasive surgery, or laparotomy), the tumor site (ovary, endometrium, cervix, vagina, and vulva), and the surgical complexity (stratified according to the Aletti score for ovarian cancer surgery and the modified score for the other pathologies). The surgical complexity was divided into three categories (low complexity, moderate complexity, and high complexity) (9). Finally, the postoperative complications data were also collected for all patients and were classified according to the Clavien Dindo classification (10) and the RIOT.

### Adaptation of the Center During the Lockdown Period

Since our center is a comprehensive cancer center, the hospital undertook the policy to maintain this a COVID-free hospital. A dedicated COVID unit was created that evaluated and tested all suspected patients and transferred them directly to the COVID units in dedicated hospitals (university hospitals). Other strategies were also employed to decrease the risk of viral contamination among the medical and non-medical staff such as the systematic use of face masks and alcohol-based hand rubs before entering the hospital. All patients underwent COVID-19 PCR testing with or without a thoracic CT scan prior to surgery. We also implemented teleconsultation as much as possible and limited family visits to the hospital to limit the risk of viral contamination. Every day during the lockdown period, a multidisciplinary medical team meeting including physicians, surgeons, anesthesiologists, and nurses took place to evaluate the extension of the pandemic and adapt the medical decisions accordingly. The fact that our center agreed to provide some of our ICU beds to severe non-COVID-19 patients coming from other hospitals induced a decrease in the availability of ICU beds and the associated decrease in the rate of complex surgery requiring postoperative ICU stay.

### Surgical Management

Since 2016, our institution has implemented the Enhanced Recovery Programs for all surgical specialties to standardize our clinical practices. The results of this implementation were previously reported (11). Our Gynecologic oncology team is made of six surgeons, of whom four are specialized in advanced ovarian cancer surgeries and advanced peritoneal carcinomatosis resections, and three are experienced in robotic-assisted surgery (cervical and endometrial cancer). Our department received the ESGO accreditation for ovarian cancer surgery in 2019, and every year, more than 50 complex ovarian cytoreductions are performed in our department.

### Postoperative Morbidity and Mortality

Medical and surgical postoperative complications were evaluated up to 30 days after surgery. Complications were defined and graded based on the Clavien Dindo classification.

### Statistical Analysis

The associations between categorical values were evaluated via the χ^2^ test. The continuous variables were analyzed with a *t*-test. Statistical significance was set as *p* ≤ 0.05. Analyses were performed with SPSS version 16.0 (SPSS Inc., Chicago, 124 Illinois).

## Results

Between January 2020 and May 2020, a total of 212 gynecologic oncology surgical procedures were performed in our surgical department. When comparing the two periods, we observed a 33% decrease in the surgical activity: 127 surgical procedures were performed in the period preceding the lockdown and 85 procedures were performed in the post-lockdown period.

The two populations presented similar clinical and epidemiologic characteristics: age, body mass index (BMI), ASA score, and tumor sites (Table 1).

**Table 1:** Description of populations and surgical procedures during the two periods.

	**Period 1 (*n* = 127)**	**Period 2 (*n* = 85)**	***p***
**Age** Mean (ST)	60.0 (14.7)	60.0 (15.9)	0.9
**BMI** Mean (ST)	25.4 (6.9)	25.3 (6.1)	0.87
**ASA score** ***n*** **(%)**			
1	24 (18.9)	20 (23.5)	
2	84 (66.1)	58 (68.2)	0.16
3	19 (15.0)	6 (7.1)	
4	0 (0)	1 (1.2)	
**Surgery** ***n*** **(%)**			
Simple	73 (57.5)	43 (50.6)	0.323
Complex	54 (42.5)	42 (49.4)	
**Organ** ***n*** **(%)**			
Ovary	51 (40.1)	43 (50.6)	
Uterus	44 (34.6)	55 (25.9)	0.143
Cervix	16 (12.6)	15 (17.6)	
Begnin	12 (9.5)	3 (3.5)	
Vulva	4 (3.2)	1 (1.2)	
Vagina	0	1 (1.2)	
**Procedure** *n* (%)			
MIS	58 (45.7)	39 (45.9)	
Laparotomy	36 (28.3)	30 (35.3)	0.592
Hysteroscopy	24 (18.9)	11 (12.9)	
Gynecologic exam	9 (7.1)	5 (5.9)	
**Aletti's score** *n* (%)			
Low	38 (70.4)	27 (64.3)	
Intermediate	15 (27.8)	10 (23.8)	0.129
high	1 (1.8)	5 (11.9)	

*BMI, Body Mass Index; ASA, American Society of Anesthesiologist score; MIS, Minimally Invasive Surgery*.

When analyzing the surgical procedures, we did not find any difference between the two compared periods of time. There was no difference concerning the type of the surgical approach (pelvic examination under anesthesia, hysteroscopy, minimally invasive surgery, and laparotomy) (*p* = 0.592), the surgical complexity (*p* = 0.129), and the perioperative (*p* = 0.791) and the postoperative complications rates (*p* = 0.102) (Tables 1, 2).

**Table 2:** Description of surgical and oncologic outcomes between the two periods.

	**Period 1 (*n* = 127)**	**Period 2 (*n* = 85)**	***p***
**LOS Mean (ST)** *All procedures* *Simple procedures* *Complex procedures*	3.0 (6.7) 0.5 (0.8) 6.4 (9.3)	3.6 (5.0)1.0 (1.5)6.3 (5.9)	0.85 0.74 0.96
**Perioperative complications** *n* (%) *None* *Clavien Dindo grade 1–2* *Clavien Dindo grade 3–4* *Clavien Dindo grade 5*	109 (85.8) 16 (12.6) 1 (0.8) 1(0.8)	73 (85.9)11 (12.9)1 (1.2)0	0.791
**Postoperative complications** *n* (%) *None* *Clavien Dindo grade 1–2* *Clavien Dindo grade 3–4* *Clavien Dindo grade 5*	123 (96.8) 1 (0.8) 3 (2.4) 0	78 (91.8)5 (5.9)2 (2.3)0	0.102
**RIOT** Mean (ST)	46.9 (20.2)	31.9 (13.6)	0.003

*LOS, Length of Stay; RIOT, Return to Intended Oncological Treatment*.

During the post-lockdown period, we observed a statistically non-significant (*p* = 0.323) decrease in the rate of simple procedures (57.5 vs. 50.6%) and an increase in the rate of complex procedures (42.5 vs. 49.4%).

The LOS was similar between the two periods (3.0 vs. 3.6 days, *p* = 0.85) and even for the complex procedure (6.4 vs. 6.3 days, *p* = 0.96).

Concerning the RIOT, we observed a statistically significant decrease (*p* = 0.003) in the RIOT in the second post-lockdown period with an average of 31.9 days (±13.6) compared with 46.9 days (±20.2) in the pre-lockdown period.

None of the patients included in the study contracted the COVID-19 infection during the hospitalization.

## Discussion

Our results suggest that applying the “care as usual” strategy during the COVID-19 pandemic is an acceptable and safe strategy to apply in comprehensive cancer centers. Our results showed a 33% decrease in surgical activity; however, this reduction concerned mainly the simple diagnostic or follow-up surgeries as it was recommended by the national and the international societies (3–5). This decrease in the activity is a result of the reorganization of the operating rooms and the ICU beds to maintain available a certain number of medical staff and ventilators in case of need (12, 13). Thus, our priority was to maintain the more urgent, more complex therapeutic procedures during the COVID-19 pandemic, and consequently, we decided to have a “care as usual “strategy during this outbreak. The preliminary Chinese experience from the Wuhan Cancer Center has shown that patients who underwent surgery for gynecologic malignancies did not present more complications (8). In our institution, since we do not have an emergency department that cares for common non-oncologic urgent pathologies, we have created and dedicated a clinical pathway that manages patients in the case of medical or surgical complications during the postoperative period. Since our institution is a comprehensive cancer center and all our patients are treated for malignancies that induce a certain immunosuppressive state and are more prone to severe infection, we decided not to accept COVID-19 patients. On the other hand, upon admission of patients, if they presented signs or symptoms of hyperthermia or COVID symptoms, they were directly screened with RT-PCR and isolated in a special unit until they receive the screening results. Our study confirms the previously reported results that by applying strict measures including social distancing, alcohol hand rub, face mask, and systematic screening of the patients prior to surgery and ensuring a COVID-19 free hospital, we managed to avoid viral contamination prior to surgical procedures (14–16).

During the study period, we did not diagnose any case of COVID-19 infection. This is concordant with the findings of Glasbey et al. (17) that conclude to provide safe elective cancer surgery in the context of COVID-19 free surgical pathways. Additionally, the literature review shows that high-volume specialized cancer centers offer better care with fewer complications, reduced LOS, and reduced RIOT with better oncologic outcomes (11, 18, 19). This was proven and is highly recommended by the French and the European society of gynecologic oncology especially for ovarian cancer management (19, 20). A Danish study confirmed that even for simple robotic-assisted oncologic procedures such as hysterectomy associated with pelvic lymphadenectomy for endometrial cancer, centralized activity improves the surgical and oncologic outcomes (21). Prior to obtaining the ESGO certification for the management of advanced ovarian cancer, we developed and implemented specific patient-centered clinical pathways. This is associated with the implementation of ERAS programs for 10 years and proved to reduce our complications rate and to optimize the oncologic outcomes (11). More recently, we developed a prehabilitation program to improve even more the surgical and oncologic outcomes. Literature review shows that prehabilitation programs reduce the LOS and the complication rate (22).

RIOT is a novel metric that can be used to evaluate the quality of the oncosurgical treatment. This corresponds to the time interval between the first day of surgical hospitalization and the return to the intended medical treatment (chemotherapy or radiotherapy) (23). The implementation of ERAS programs participates in reducing the delay between surgery and adjuvant treatment with a potentially positive impact on the oncologic outcomes (24). Our findings are in accordance with Thomakos et al. (25) who emphasized that the ERAS program must be continued during the COVID-19 pandemic outbreak. In our study, during the post-lockdown period, the RIOT was 16 days shorter compared to the RIOT in the pre-lockdown period. These results may be explained by the better implementation of patient-centered clinical pathways and better communication between the medical team members (surgeons, oncologists, and radiation oncologists) due to the less important clinical activity during the pandemic period compared to the pre-lockdown period. Finally, since it was uncertain whether the clinical and surgical activity (including chemotherapy and radiotherapy) was going to be maintained, an important effort was made to anticipate and organize patients' clinical pathways. All the three previously cited factors have certainly participated to reduce the RIOT in our institution, which is finally a very important point to underline in this context of the COVID-19 pandemic. The “care as usual” strategy did not increase our complications rate, our LOS, and our COVID infection rate.

Another factor that participated in maintaining the gynecologic oncologic activity is the fact that other specialties have decreased their activity such as the senology department. All prophylactic and reconstructive breast surgeries were postponed. Furthermore, the cessation of breast cancer screening during the pandemic induced a delay in the management of occult breast cancers (26). In outbreak time, the multidisciplinarity to maintain surgical activity is another strength.

## Conclusion

In conclusion, we think that even during the pandemic period, gynecologic oncology activity must be maintained in order not to deprive cancer patients to be treated adequately. Even though our study is based on a small retrospective cohort, we think that the “care as usual” strategy seems an acceptable approach to adopt during the pandemic period in a high-volume gynecologic oncology surgical department. We believe that this strategy may be integrated in the future recommendations, because it has proven to maintain surgical activity and good oncologic outcomes even in the exceptional pandemic context.

## Data Availability Statement

The original contributions presented in the study are included in the article/supplementary material, further inquiries can be directed to the corresponding author/s.

## Ethics Statement

The studies involving human participants were reviewed and approved by Paoli Calmettes Institut review board. The patients/participants provided their written informed consent to participate in this study.

## Author Contributions

GB, HE, CJ, GH, LS, JB, IM, JL, DM, MF, and EL: conceptualization, methodology, writing—original draft, and writing—review and editing. All authors contributed to the article and approved the submitted version.

## Conflict of Interest

The authors declare that the research was conducted in the absence of any commercial or financial relationships that could be construed as a potential conflict of interest.
